# Nucleophilic Aromatic Substitution of Polyfluoroarene to Access Highly Functionalized 10-Phenylphenothiazine Derivatives

**DOI:** 10.3390/molecules26051365

**Published:** 2021-03-04

**Authors:** Kotaro Kikushima, Haruka Koyama, Kazuki Kodama, Toshifumi Dohi

**Affiliations:** College of Pharmaceutical Sciences, Ritsumeikan University, 1-1-1 Nojihigashi, Kusatsu, Shiga 525–8577, Japan; ph0104eh@ed.ritsumei.ac.jp (H.K.); ph0112hv@ed.ritsumei.ac.jp (K.K.)

**Keywords:** polyfluoroarene, phenothiazine, nucleophilic aromatic substitution, amination, photocatalyst

## Abstract

Nucleophilic aromatic substitution (S_N_Ar) reactions can provide metal-free access to synthesize monosubstituted aromatic compounds. We developed efficient S_N_Ar conditions for *p*-selective substitution of polyfluoroarenes with phenothiazine in the presence of a mild base to afford the corresponding 10-phenylphenothiazine (PTH) derivatives. The resulting polyfluoroarene-bearing PTH derivatives were subjected to a second S_N_Ar reaction to generate highly functionalized PTH derivatives with potential applicability as photocatalysts for the reduction of carbon–halogen bonds.

## 1. Introduction

Owing to the high electronegativity of fluorine atoms, polyfluoroarenes can undergo nucleophilic aromatic substitution (S_N_Ar) [1], wherein nucleophiles attack the low-electron-density arene core, and the fluoride anion is eliminated as a fluoride salt. Although transition-metal-catalyzed C-F and C-H bond functionalization of polyfluoroarenes have advanced considerably in recent years [2,3,4,5,6], S_N_Ar of polyfluoroarenes offers a transition-metal-free approach to substituted polyfluoroarenes. Polyfluoroarenes react with organometallic compounds, such as organolithium or organomagnesium reagents, to convert aromatic C-F bonds into C-C bonds without the use of transition metal catalysts [7,8]. The combination of a fluoride salt and organosilane compounds as nucleophiles has also been successful in the S_N_Ar of polyfluoroarenes, wherein the reaction proceeds with a catalytic amount of a fluoride anion [9,10,11,12,13]. The use of alcohols or amines as nucleophiles enables C-O and C-N bond formation to produce the corresponding aryl ether and aniline derivatives [8,14,15].

Functionalized arenes, such as arylamine derivatives, can be synthesized via transition-metal-catalyzed cross-coupling reactions [16,17,18,19,20,21]. However, the high cost of organometallic catalysts and contamination of the resulting products with metal traces represent major drawbacks of such methods. Alternative methods for transition-metal-free synthesis of arylamine derivatives have been achieved using hypervalent iodine reagents [22,23,24,25,26,27], sulfonium reagents [28], nitorarenes [29,30,31,32,33], or electrochemical conditions [34]. The S_N_Ar reaction also offers an alternative method without the use of transition metals, which is therefore potentially applicable in the facile synthesis of organic functional materials containing substituted arenes. For instance, 1,2,3,5-tetrakis(carbazolyl)-4,6-dicyanobenzene (4CzIPN), an organic photocatalyst, has been produced by multiple S_N_Ar reactions using carbazole and 1,3-dicyano-2,4,5,6-terafluorobenzene with NaH as the base [35]. In this context, we focused on the application of a sequential and controllable S_N_Ar reaction for the synthesis of 10-phenylphenothiazine (PTH) derivatives, which serve as organic photocatalysts that induce dehalogenative bond formation via processes such as atom transfer radical polymerization [36,37,38,39,40,41,42,43,44,45,46].

**Scheme 1:**
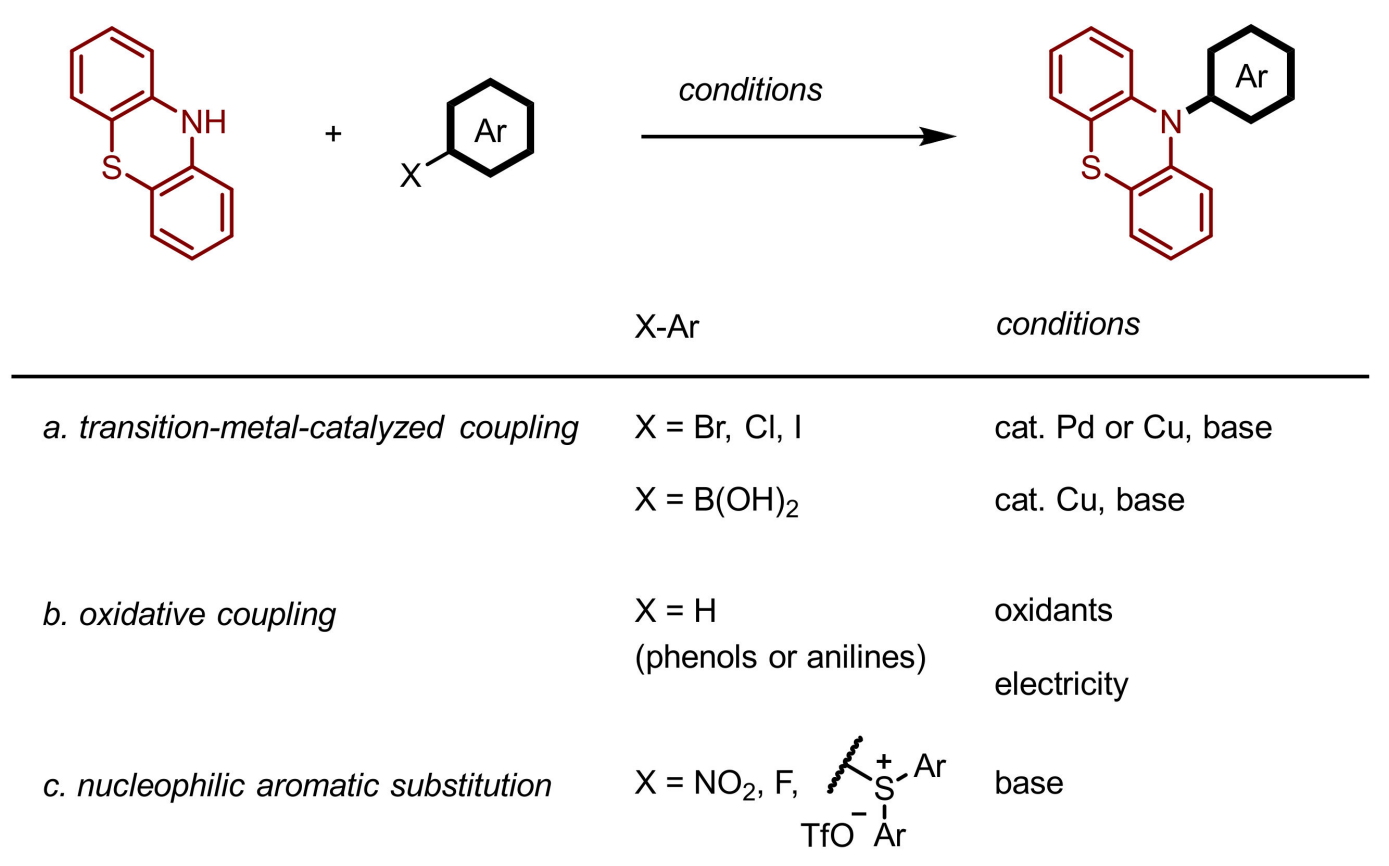
Access to PTH derivatives under various conditions.

PTH derivatives are generally prepared via palladium- or copper-catalyzed coupling reactions of phenothiazine with aryl halides or arylboronic acids (Scheme 1a) [36,37,38,39,40,41,42,43,44,45,46,47,48,49,50,51,52]. Transition-metal-free methods for the synthesis of PTH derivatives include oxidative C-H amination in the presence of oxidants [53,54,55,56,57] or under electrolytic conditions [58,59], although starting materials are limited to phenols and anilines (Scheme 1b). In addition, S_N_Ar of triphenylsulfonium salts, nitroarenes, and fluoroarenes with phenothiazine have been demonstrated for the preparation of PTH derivatives (Scheme 1c) [28,60,61,62,63,64,65,66,67].

**Scheme 2:**
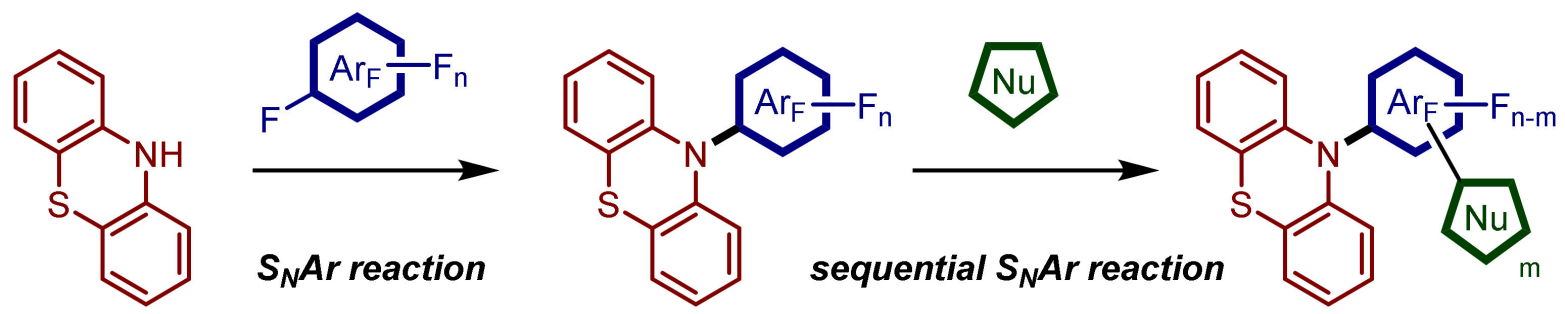
Synthesis of highly functionalized PTH derivatives via sequential S_N_Ar reactions.

We envisioned that a reaction between phenothiazine and polyfluoroarenes would afford the corresponding polyfluoroarene-bearing PTH derivatives, which would then undergo a second S_N_Ar reaction for the introduction of other nucleophiles to afford highly functionalized PTH derivatives (Scheme 2). In addition to the synthetic utility of polyfluoroarenes, their introduction provides unique functionalities crucial in materials science, such as improvement of oxidation resistance, lowering of both HOMO and LUMO energy levels, and favorable stacking interactions with electron-rich aromatic rings [68,69,70]. However, S_N_Ar of polyfluoroarenes often suffers from uncontrollable substitution, resulting in a mixture of regioisomers and/or multisubstituted products. Therefore, it is necessary to establish appropriate conditions that suppress unselective substitution events and over-reactions, while being applicable to a wide range of polyfluoroarenes. Herein, we demonstrate S_N_Ar of various polyfluoroarenes resulting in mono-phenothiazination in the presence of an appropriate base to afford PTH derivatives bearing polyfluoroarenes, and further transformation of the resulting PTH derivatives to highly functionalized PTH derivatives via a second S_N_Ar reaction.

## 2. Results and Discussion

**Scheme 3:**
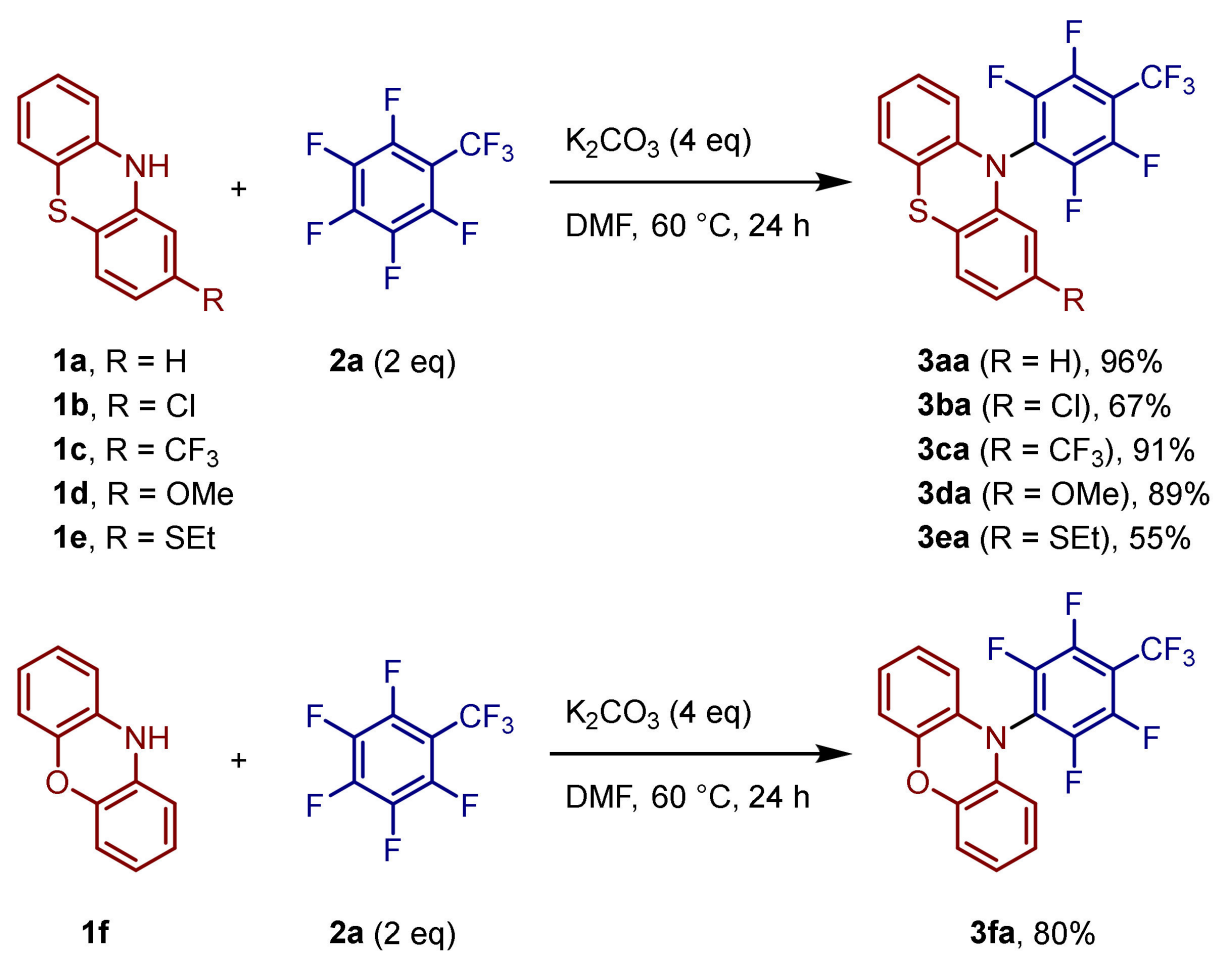
S_N_Ar reaction of octafluorotoluene with phenothiazine derivatives or phenoxazine.

The reaction of phenothiazine with octafluorotoluene in the presence of K_2_CO_3_ in *N*,*N*-dimethylformamide (DMF) at 60 °C afforded the corresponding PTH derivative **3aa** as the sole product in 96% yield (Scheme 3). The fluorine atom at the *p*-position of the trifluoromethyl group of octafluorotoluene was substituted by phenothiazine, without the formation of regioisomers or multisubstituted products. The observed regioselectivity was in agreement with previously reported outcomes of octafluorotoluene S_N_Ar [11,13], and it is governed by the electron density at the reactive carbons (*ortho*- and *para*-positions) on the aromatic ring and the steric repulsion between the trifluoromethyl group and bulky phenothiazine. The K_2_CO_3_/DMF system was found to be an efficient combination for mono S_N_Ar between various phenothiazines and octafluorotoluene (Scheme 3). For example, phenothiazine derivatives bearing electron-deficient and electron-donating groups (**1b**–**1e**) were employed in the present reaction to give the corresponding PTH derivatives (**3ba**–**3ea**). Moreover, phenoxazine derivative **3fa** was synthesized under similar conditions. Next, we examined various polyfluoroarenes for the S_N_Ar reaction with phenothiazine.

**Scheme 4:**
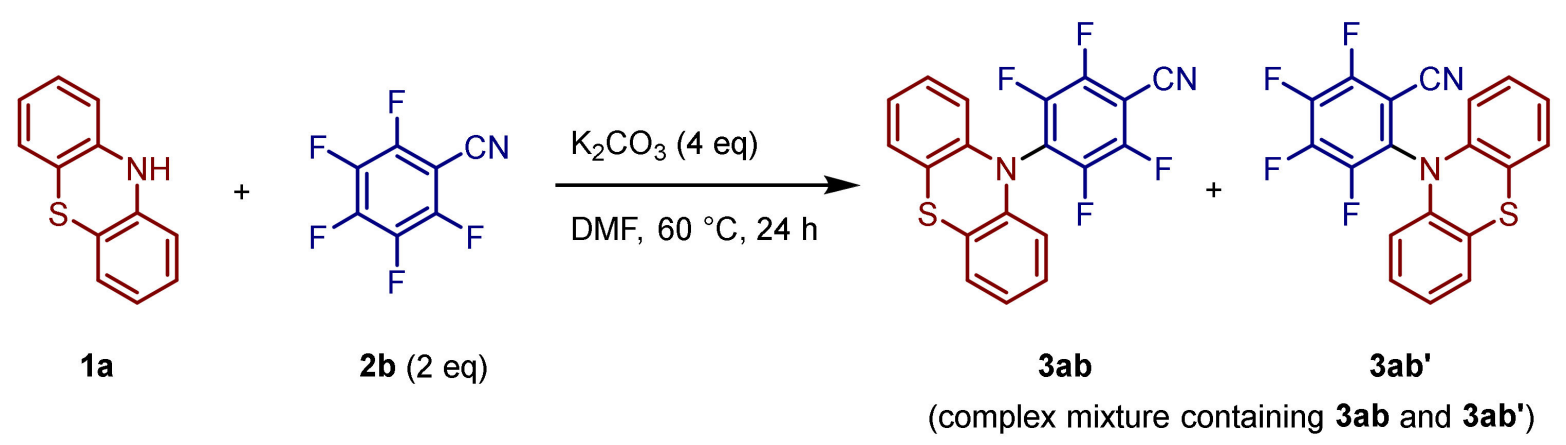
Reaction of pentafluorobenzonitrile with phenothiazine using the K_2_CO_3_/DMF system.

In contrast to octafluorotoluene, several other polyfluoroarenes exhibited decreased selectivities with the combination of K_2_CO_3_ and DMF, due to their inherently high reactivities, and the reaction of pentafluorobenzonitrile yielded complex mixtures including *p*- and *o*-substituted products, **3ab** and **3ab’**, respectively (Scheme 4). Pentafluoronitrobenzene provided similar results, undergoing uncontrollable S_N_Ar.

Thus, optimization of the reaction conditions was performed for pentafluorobenzonitrile (**2b**) to suppress multiple substitution (Table 1). Using Li_2_CO_3_ or Na_2_CO_3_ instead of K_2_CO_3_ afforded the desired product **3ab** in low yield along with unreacted **2b** (entries 1 and 2). On the other hand, the use of Cs_2_CO_3_ led to high reactivity, and multiple substitutions occurred to give a complex mixture, containing **3ab** in 13% (entry 3). Inorganic phosphate salts, such as Li_3_PO_4_ and Na_3_PO_4_, exhibited comparable results to those of carbonate salts (entries 4 and 5). On the other hand, the use of K_3_PO_4_ improved the reaction yield of **3ab** to 48% (entry 6). The use of Na_3_PO_4_ or K_3_PO_4_ at an elevated reaction temperature of 80 °C resulted in lower yields compared to those attained under the conditions in entry 6 (entries 7 and 8). Next, we surveyed reaction solvents. In the case of acetonitrile (MeCN) at 60 °C, the reaction yield improved to 76% (entry 9). *N*,*N*-Dimethylacetoamide (DMA) and dimethyl sulfoxide (DMSO) were also suitable, albeit providing slightly decreased yields (entries 10 and 11). Chloroform, tetrahydrofuran (THF), and 1,4-dioxane were found to be inappropriate solvents (entries 12–14).

**Scheme 5:**
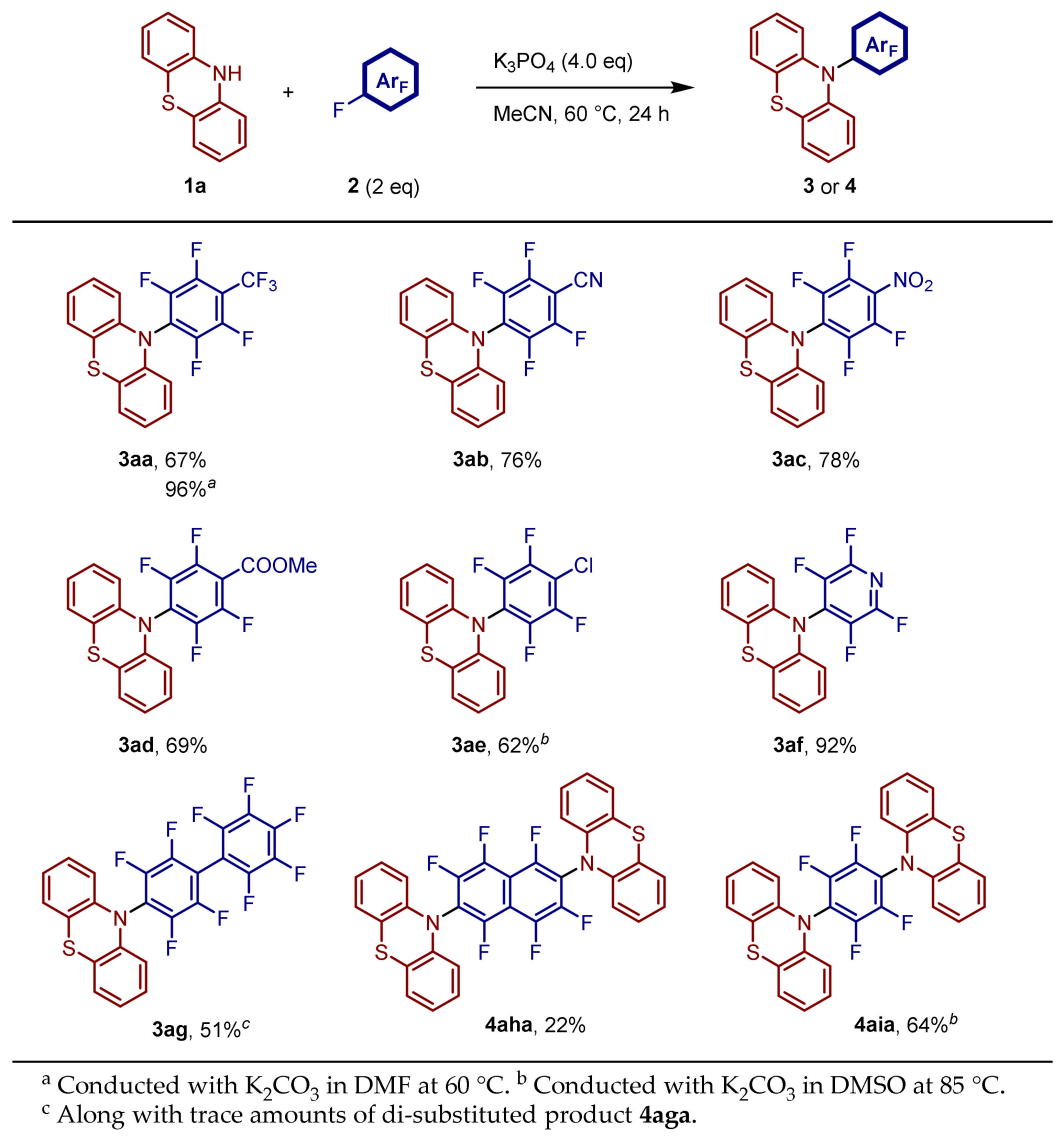
S_N_Ar reaction of phenothiazine with various polyfluoroarenes.

Next, various polyfluoroarenes were subjected to S_N_Ar with phenothiazine under the optimum conditions of K_3_PO_4_ in MeCN at 60 °C, as summarized in Scheme 5. Under these conditions, octafluorotoluene (**2a**) produced **3aa** in 67% yield, which was lower than that obtained with the use of K_2_CO_3_ and DMF. Pentafluoronitrobenzene (**2c**) also underwent S_N_Ar with high selectivity to afford *p*-substituted product **3ac** in 78% yield. Ester-bearing PTH derivative **3ad** was synthesized from methyl pentafluorobenzoate (**2d**) in 69% yield. Thus, the combination of K_3_PO_4_ and MeCN proved effective for achieving *p*-selective mono-substitution of a wide range of highly reactive polyfluoroarenes. Chloropentafluorobenzene (**2e**) underwent S_N_Ar using K_2_CO_3_ in DMSO at 85 °C to afford the corresponding product **3ae**, while the K_3_PO_4_/MeCN system resulted in low yield. The use of DMSO improved the reactivity of substitution presumably due to the higher solubility of the base. It should be noted that selective C–F bond functionalization occurred and the chlorine atom remained intact under these S_N_Ar conditions, allowing for further product transformation via transition-metal-catalyzed cross-coupling reactions. In contrast to results obtained with electron-deficient groups, methyl-substituted pentafluorobenzene did not furnish the desired product even under K_2_CO_3_/DMSO conditions. When pentafluoropyridine (**2f**) was employed as the substrate, the S_N_Ar reaction proceeded smoothly under K_3_PO_4_/MeCN conditions to produce fluorinated pyridylphenothiazine **3af** in 92% yield. Simple polyfluoroarenes lacking other functional groups were also tested in the present S_N_Ar protocol. The reaction of decafluorobiphenyl (**2g**) afforded the corresponding mono-substituted product **3ag** in 51% yield, along with a trace amount of the disubstituted compound (**4aga**). On the other hand, octafluoronaphthalene (**2h**) underwent double substitution to give **4aha** in 22% yield, even with 2 equivalents of **2h**. Hexafluorobenzene (**2i**) exhibited low reactivity under the K_3_PO_4_/MeCN system, as was the case with **2e**. The combination of K_2_CO_3_ and DMSO at 85 °C led to double substitution of **2i** affording **4aia** in 64% yield. In this case, **2i** exists in the vapor phase as a result of its low boiling point (bp: ca. 80 °C); therefore, once the first S_N_Ar reaction occurs, the second is favored due to the monosubstituted product being in solution while the bulk of **2i** remains in the vapor phase.

**Scheme 6:**
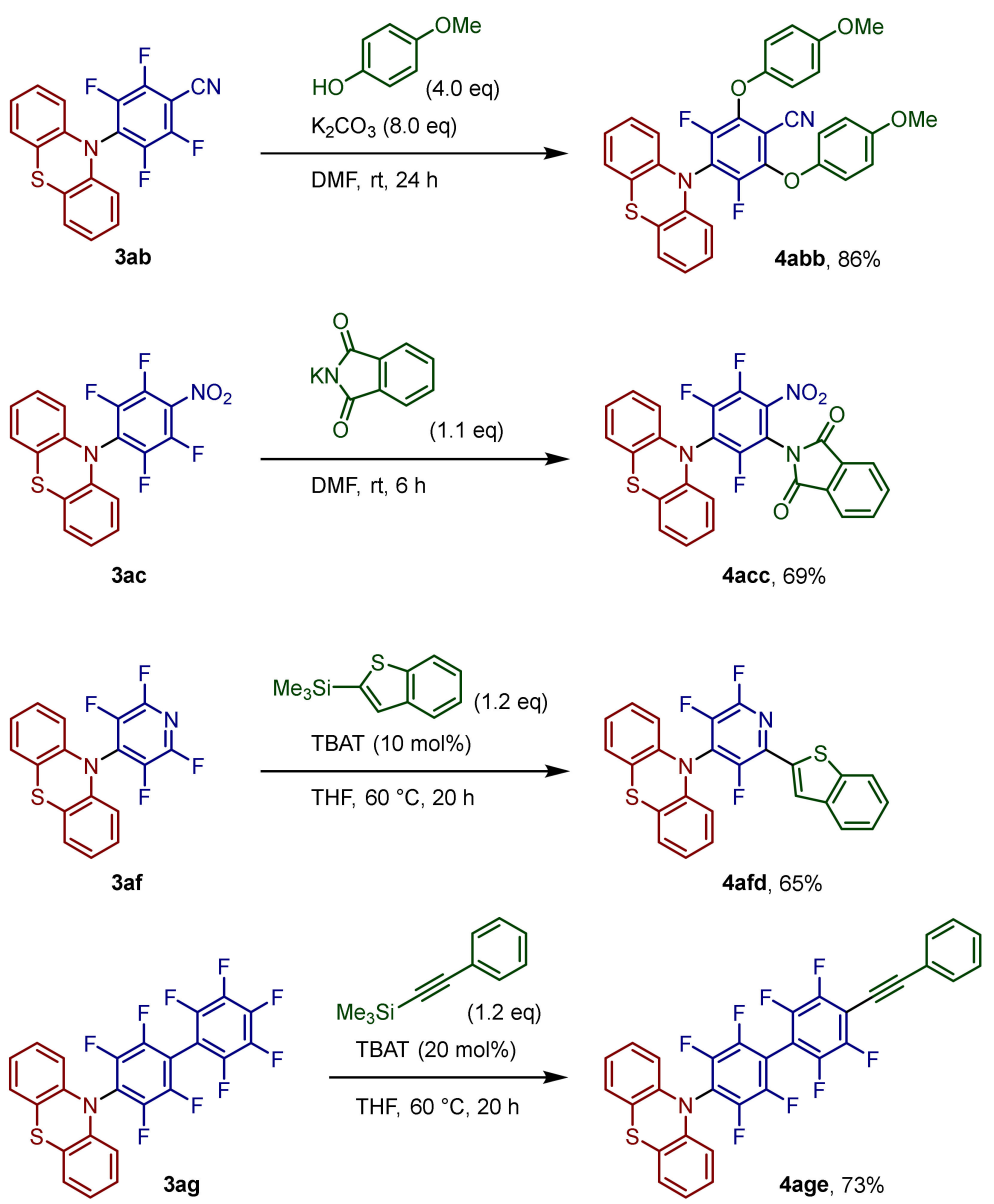
Synthesis of highly functionalized PTH derivatives via S_N_Ar.

Next, further transformations of obtained PTH derivatives **3** were performed (Scheme 6). Thus, S_N_Ar of **3ab** with *p*-methoxyphenol proceeded in the presence of K_2_CO_3_ to afford PTH derivative **4abb**, bearing both cyano and phenoxy groups. Phthalimide, commonly used as a protecting group and photosensitizer, was also introduced onto **3ac** via further S_N_Ar to obtain multifunctionalized **4acc**. Transition-metal-free carbon–carbon bond formation was also examined using a combination of organosilanes and a catalytic amount of Bu_4_NSiF_2_Ph_3_ (TBAT). Thiophene moieties, ubiquitous in functional organic materials owing to their high electron density, can be introduced onto **3af** via the reaction with thienyl silane and TBAT to afford diheteroaromatic **4afd**. Similarly, ethynylsilane participated in the carbon–carbon bond forming reaction with **3ag** to produce linear analog **4age**. Hence, PTH derivatives bearing various functional groups, connected through C–O, C–N, and C–C bonds, were synthesized via sequential S_N_Ar of polyfluoroarenes under transition-metal-free conditions.

## 3. Materials and Methods

### 3.1. General Information

^1^H, ^13^C, and ^19^F nuclear magnetic resonance (NMR) spectra were recorded on a JEOL JMN-400 spectrometer at 25 °C unless otherwise noted. The data are reported as follows: chemical shift in part per million (δ), multiplicity (s = singlet, d = doublet, t = triplet, q = quartet, and m = multiplet), integration, and coupling constant (Hz). The chemical shifts in the ^1^H NMR spectra were recorded relative to the residual solvent peaks (CDCl_3_: δ 7.26). The chemical shifts in the ^13^C NMR spectrum were also recorded relative to the residual solvent peaks (CDCl_3_: δ 77.0). The chemical shifts in the ^19^F NMR spectrum were recorded relative to that of the internal standard (4-fluorotoluene: δ −121.0). High-resolution mass spectra (HRMS) were obtained using a Thermo Scientific Exactive Plus Orbitrap (Thermo Fisher Scientific, Inc., Waltham, MA, USA). All commercially available reagents were used as received unless otherwise noted.

### 3.2. S_N_Ar Reaction of Phenothiazines with Polyfluoroarenes

#### 3.2.1. General Procedure A for the Reaction of Phenothiazines with Polyfluoroarenes

Phenothiazine derivatives (1.0 mmol) and base (4.0 mmol, 4.0 eq) were placed in a screw-capped test tube and dried under vacuum for 1 h. After backfilling with N_2_, solvent (10 mL) and polyfluoroarenes (2.1 mmol, 2.1 eq) were added in this order. The reaction mixture was stirred at 60 °C for 24 h. The reaction was quenched with water (50 mL), and the mixture was transferred to a separatory funnel containing diethyl ether (50 mL). The organic layer was separated, and the aqueous layer was extracted with diethyl ether (2 × 20 mL). The combined organic fractions were washed with brine (50 mL), dried over Na_2_SO_4_, and all volatiles were removed under vacuum. The residue was purified by flash column chromatography (SiO_2_) to yield the corresponding 10-phenylphenothiazine (PTH) derivatives.

#### 3.2.2. 10-(2,3,5,6-Tetrafluoro-4-(trifluoromethyl)phenyl)-10*H*-phenothiazine (**3aa**)

The title compound was prepared according to General Procedure A with phenothiazine (**1a**, 200 mg, 1.0 mmol), octafluorotoluene (**2a**, 300 μL, 2.1 mmol, 2.1 eq), and K_2_CO_3_ (554 mg, 4.0 mmol, 4.0 eq) in DMF (10 mL). **3aa** was isolated by flash column chromatography (SiO_2_, AcOEt/hexane = 1/100) in 96% yield (398 mg, 0.958 mmol) as a pale yellow solid. ^1^H NMR (400 MHz, CDCl_3_, rt, δ/ppm): 7.12 (dd, *J* = 7.3, 2.0 Hz, 2H), 6.93–7.02 (m, 4H), 6.26 (dd, *J* = 7.8, 1.5 Hz, 2H). ^19^F NMR (376 MHz, CDCl_3_, rt, δ/ppm): −58.5 (t, *J* = 22.0 Hz, 3F), −140.5–(−140.6) (m, 2F), −142.0–(−142.1) (m, 2F). HRMS (DART) *m/z*: ([M + H]^+^) Calcd for C_19_H_9_F_7_NS 416.0338; Found 416.0342.

#### 3.2.3. 2-Chloro-10-(2,3,5,6-tetrafluoro-4-(trifluoromethyl)phenyl)-10*H*-phenothiazine (**3ba**)

The title compound was prepared according to General Procedure A with 2-chloro-10*H*-phenothiazine (**1b**, 66.9 mg, 0.50 mmol), octafluorotoluene (**2a**, 150 μL, 1.05 mmol, 2.1 eq), and K_2_CO_3_ (277 mg, 2.0 mmol, 4.0 eq) in DMF (5 mL). **3ba** was isolated by flash column chromatography (SiO_2_, AcOEt/hexane = 1/200) in 67% yield (150 mg, 0.334 mmol) as a pale yellow solid. ^1^H NMR (400 MHz, CDCl_3_, rt, δ/ppm): 7.11 (dd, *J* = 7.3, 2.0 Hz, 1H), 7.04–6.93 (m, 4H), 6.27–6.23 (m, 2H). ^19^F NMR (376 MHz, CDCl_3_, rt, δ/ppm): −58.5 (t, *J* = 22.0 Hz, 3F), −140.4–(−140.6) (m, 2F), −142.1–(−142.2) (m, 2F). HRMS (DART) *m/z*: ([M + H]^+^) Calcd for C_19_H_8_ClF_7_NS^+^ 449.9949; Found 449.9946.

#### 3.2.4. 10-(2,3,5,6-Tetrafluoro-4-(trifluoromethyl)phenyl)-2-(trifluoromethyl)- 10*H*-phenothiazine (**3ca**)

The title compound was prepared according to General Procedure A with 2-(trifluoromethyl)-10*H*-phenothiazine (**1c**, 133.6 mg, 0.50 mmol), octafluorotoluene (**2a**, 150 μL, 1.05 mmol, 2.1 eq), and K_2_CO_3_ (277 mg, 2.0 mmol, 4.0 eq) in DMF (5 mL). **3ca** was isolated by flash column chromatography (SiO_2_, AcOEt/hexane = 1/200) in 91% yield (220 mg, 0.455 mmol) as a pale yellow solid. ^1^H NMR (400 MHz, CDCl_3_, rt, δ/ppm): 7.21 (s, 2H), 7.13–7.10 (m, 1H), 7.06–6.98 (m, 2H), 6.43 (s, 1H), 6.27 (d, *J* = 7.3 Hz, 1H). ^19^F NMR (376 MHz, CDCl_3_, rt, δ/ppm): −58.5 (t, *J* = 22.0 Hz, 3F), −65.1 (s, 3F), −139.3–(−139.7) (m, 2F), −142.1–(−142.2) (m, 2F). HRMS (DART) *m/z*: ([M + H]^+^) Calcd for C_20_H_8_F_10_NS^+^ 484.0212; Found 484.0210.

#### 3.2.5. 2-Methoxy-10-(2,3,5,6-tetrafluoro-4-(trifluoromethyl)phenyl)-10*H*-phenothiazine (**3da**)

The title compound was prepared according to General Procedure A with 2-methoxy-10*H*-phenothiazine (**1d**, 114.6 mg, 0.50 mmol), octafluorotoluene (**2a**, 150 μL, 1.05 mmol, 2.1 eq), and K_2_CO_3_ (277 mg, 2.0 mmol, 4.0 eq) in DMF (5 mL). **3da** was isolated by flash column chromatography (SiO_2_, AcOEt/hexane = 1/150) in 89% yield (200 mg, 0.449 mmol) as a pale yellow solid. ^1^H NMR (400 MHz, CDCl_3_, rt, δ/ppm): 7.05 (dd, *J* = 7.8, 2.0 Hz, 1H), 6.96 (d, *J* = 8.3 Hz, 1H), 6.93–6.87 (m, 2H), 6.46 (d, *J* = 7.3 Hz, 1H), 6.20–6.17 (m, 1H), 5.79–5.77 (m, 1H), 3.64 (s, 3H). ^19^F NMR (376 MHz, CDCl_3_, rt, δ/ppm): −58.5 (t, *J* = 22.0 Hz, 3F), −139.7–(−140.0) (m, 2F), −142.1–(−142.2) (m, 2F). HRMS (DART) *m/z*: ([M + H]^+^) Calcd for C_20_H_11_F_7_NS^+^ 446.0444; Found 446.0447.

#### 3.2.6. 2-(Ethylthio)-10-(2,3,5,6-tetrafluoro-4-(trifluoromethyl)phenyl)-10*H*-phenothiazine (**3ea**)

The title compound was prepared according to General Procedure A with 2-ethylthio-10*H*-phenothiazine (**1e**, 129.7 mg, 0.50 mmol), octafluorotoluene (**2a**, 150 μL, 1.05 mmol, 2.1 eq), and K_2_CO_3_ (277 mg, 2.0 mmol, 4.0 eq) in DMF (5 mL). **3ea** was isolated by flash column chromatography (SiO_2_, AcOEt/hexane = 1/100) in 55% yield (130 mg, 0.273 mmol) as a yellow oil. ^1^H NMR (400 MHz, CDCl_3_, rt, δ/ppm): 7.10–7.08 (m, 1H), 7.03–6.91 (m, 4H), 6.25–6.21 (m, 2H), 2.81 (q, *J* = 7.3 Hz, 2H), 1.23 (t, *J* = 7.3 Hz, 3H). ^19^F NMR (376 MHz, CDCl_3_, rt, δ/ppm): −58.5 (t, *J* = 19.5 Hz, 3F), −140.2–(−140.4) (m, 2F), −142.1–(−142.2) (m, 2F). HRMS (DART) *m/z*: ([M + H]^+^) Calcd for C_21_H_13_F_7_NS_2_^+^ 476.0372; Found 476.0371.

#### 3.2.7. 10-(2,3,5,6-Tetrafluoro-4-(trifluoromethyl)phenyl)-10*H*-phenoxazine (**3fa**)

The title compound was prepared according to General Procedure A with 10*H*-phenoxazine (**1f**, 91.6 mg, 0.50 mmol), octafluorotoluene (**2a**, 150 μL, 1.05 mmol, 2.1 eq), and K_2_CO_3_ (277 mg, 2.0 mmol, 4.0 eq) in DMF (5 mL). **3fa** was isolated by flash column chromatography (SiO_2_, AcOEt/hexane = 1/100) in 80% yield (160 mg, 0.400 mmol) as a pale yellow solid. ^1^H NMR (400 MHz, CDCl_3_, rt, δ/ppm): 6.82–6.78 (m, 4H), 6.75–6.69 (m, 2H), 6.00 (dd, *J* = 7.3, 1.5 Hz, 2H). ^19^F NMR (376 MHz, CDCl_3_, rt, δ/ppm): −58.5 (t, *J* = 22.0 Hz, 3F), −140.3–(−140.5) (m, 2F), −142.0–(−142.1) (m, 2F). HRMS (DART) *m/z*: ([M + H]^+^) Calcd for C_19_H_9_F_7_ON^+^ 400.0567; Found 400.0565.

#### 3.2.8. 2,3,5,6-Tetrafluoro-4-(10*H*-phenothiazin-10-yl)benzonitrile (**3ab**)

The title compound was prepared according to General Procedure A with phenothiazine (**1a**, 399 mg, 2.0 mmol), pentafluorobenzonitrile (**2b**, 512 μL, 4.0 mmol, 2.0 eq), and K_3_PO_4_ (1.70 g, 8.0 mmol, 4.0 eq) in MeCN (20 mL). **3ab** was isolated by flash column chromatography (SiO_2_, AcOEt/hexane = 1/40) in 76% yield (568 mg, 1.53 mmol) as a pale yellow solid. ^1^H NMR (400 MHz, CDCl_3_, rt, δ/ppm): 7.13 (dd, *J* = 7.3, 2.0 Hz, 2H), 7.08–6.95 (m, 4H), 6.27 (dd, *J* = 7.8, 1.5 Hz, 2H). ^19^F NMR (376 MHz, CDCl_3_, rt, δ/ppm): −132.4–(−132.5) (m, 2F), −140.6–(−140.7) (m, 2F). HRMS (DART) *m/z*: ([M + H]^+^) Calcd for C_19_H_9_F_4_N_2_S 373.0417; Found 373.0415.

#### 3.2.9. 10-(2,3,5,6-Tetrafluoro-4-nitrophenyl)-10*H*-phenothiazine (**3ac**)

The title compound was prepared according to General Procedure A with phenothiazine (**1a**, 399 mg, 2.0 mmol), pentafluoronitrobenzene (**2c**, 496 μL, 4.0 mmol, 2.0 eq), and K_3_PO_4_ (1.70 g, 8.0 mmol, 4.0 eq) in MeCN (20 mL). **3ac** was isolated by flash column chromatography (SiO_2_, hexane) in 78% yield (613 mg, 1.56 mmol) as an orange solid. ^1^H NMR (400 MHz, CDCl_3_, rt, δ/ppm): 7.11 (dd, *J* = 7.3, 1.5 Hz, 2H), 7.02–6.94 (m, 4H), 6.26 (dd, *J* = 7.3, 1.5 Hz, 2H). ^19^F NMR (376 MHz, CDCl_3_, rt, δ/ppm): −140.1–(−140.1) (m, 2F), −146.9–(−146.9) (m, 2F). HRMS (DART) *m/z*: ([M + H]^+^) Calcd for C_18_H_9_F_4_N_2_O_2_S^+^ 393.0315; Found 393.0317.

#### 3.2.10. Methyl 2,3,5,6-tetrafluoro-4-(10*H*-phenothiazin-10-yl)benzoate (**3ad**)

The title compound was prepared according to General Procedure A with phenothiazine (**1a**, 100 mg, 0.50 mmol), methyl pentafluorobenzoate (**2d**, 146 μL, 1.0 mmol, 2.0 eq), and K_3_PO_4_ (424.5 mg, 2.0 mmol, 4.0 eq) in MeCN (5 mL). **3ad** was isolated by flash column chromatography (SiO_2_, AcOEt/hexane = 1/200) in 69% yield (140 mg, 0.345 mmol) as a pale yellow solid. ^1^H NMR (400 MHz, CDCl_3_, rt, δ/ppm): 7.10 (dd, *J* = 7.3, 1.5 Hz, 2H), 7.00–6.91 (m, 4H), 6.25 (dd, *J* = 7.8, 1.0 Hz, 2H), 4.05 (s, 3H). ^19^F NMR (376 MHz, CDCl_3_, rt, δ/ppm): −143.2–(−143.3) (m, 2F), −139.7–(−139.8) (m, 2F). HRMS (DART) *m/z*: ([M + H]^+^) Calcd for C_20_H_12_F_4_NO_2_S^+^ 406.0519; Found 406.0520.

#### 3.2.11. 10-(4-Chloro-2,3,5,6-tetrafluorophenyl)-10*H*-phenothiazine (**3ae**)

The title compound was prepared according to General Procedure A with phenothiazine (**1a**, 100 mg, 0.50 mmol), chloropentafluorobenzene (**2e**, 129 μL, 1.0 mmol, 2.0 eq), and K_2_CO_3_ (277 mg, 2.0 mmol, 4.0 eq) in DMSO (5 mL) at 80 °C. **3ae** was isolated by flash column chromatography (SiO_2_, AcOEt/hexane = 1/200) in 62% yield (118 mg, 0.309 mmol) as a pale yellow solid. ^1^H NMR (400 MHz, CDCl_3_, rt, δ/ppm): 7.09 (dd, *J* = 7.3, 1.5 Hz, 2H), 7.00–6.91 (m, 4H), 6.26 (dd, *J* = 7.8, 1.5 Hz, 2H). ^19^F NMR (376 MHz, CDCl_3_, rt, δ/ppm): −141.0–(−141.0) (m, 2F), −143.8–(−143.8) (m, 2F). HRMS (DART) *m/z*: ([M + H]^+^) Calcd for C_18_H_9_ClF_4_NS^+^ 382.0075; Found 382.0075.

#### 3.2.12. 10-(Perfluoropyridin-4-yl)-10*H*-phenothiazine (**3af**)

The title compound was prepared according to General Procedure A with phenothiazine (**1a**, 399 mg, 2.0 mmol), pentafluoropyridine (**2f**, 430 μL, 4.0 mmol, 2.0 eq), and K_3_PO_4_ (1.70 g, 8.0 mmol, 4.0 eq) in MeCN (20 mL). **3af** was isolated by flash column chromatography (SiO_2_, AcOEt/hexane = 1/50) in 92% yield (640 mg, 1.84 mmol) as a pale yellow solid. ^1^H NMR (400 MHz, CDCl_3_, rt, δ/ppm): 7.06 (dd, *J* = 6.8, 1.5 Hz, 2H), 7.04–6.94 (m, 4H), 6.34 (d, *J* = 7.8 Hz, 2H). ^19^F NMR (376 MHz, CDCl_3_, rt, δ/ppm): −88.9–(−89.0) (m, 2F), −144.5–(−144.7) (m, 2F). HRMS (DART) *m/z*: ([M + H]^+^) Calcd for C_17_H_9_F_4_N_2_S^+^ 349.0417; Found 349.0420.

#### 3.2.13. 10-(Perfluoro-[1,1’-biphenyl]-4-yl)-10*H*-phenothiazine (**3ag**)

The title compound was prepared according to General Procedure A with phenothiazine (**1a**, 100 mg, 0.50 mmol), decafluorobiphenyl (**2g**, 334 mg, 1.0 mmol, 2.0 eq), and K_3_PO_4_ (424.5 mg, 2.0 mmol, 4.0 eq) in MeCN (5 mL). **3ag** was isolated by flash column chromatography (SiO_2_, AcOEt/hexane = 1/100) in 51% yield (130 mg, 0.253 mmol) as a pale yellow solid. ^1^H NMR (400 MHz, CDCl_3_, rt, δ/ppm): 7.10 (dd, *J* = 7.8, 1.5 Hz, 2H), 7.03–6.92 (m, 4H), 6.33 (d, *J* = 7.8 Hz, 2H). ^19^F NMR (376 MHz, CDCl_3_, rt, δ/ppm): −138.4–(−138.4) (m, 2F), −139.1–(−139.2) (m, 2F), −143.4–(−143.5) (m, 1F), −151.4–(−151.6) (m, 2F), −162.2–(−162.3) (m, 2F). HRMS (DART) *m/z*: ([M + H]^+^) Calcd for C_24_H_9_F_9_NS^+^ 514.0307; Found 514.0303.

#### 3.2.14. 10,10’-(Perfluoronaphthalene-2,6-diyl)bis(10*H*-phenothiazine) (**4aha**)

The title compound was prepared according to General Procedure A with phenothiazine (**1a**, 100 mg, 0.50 mmol), octafluoronaphthalene (**2h**, 270 mg, 1.0 mmol, 2.0 eq), and K_3_PO_4_ (424.5 mg, 2.0 mmol, 4.0 eq) in MeCN (5 mL). **4aha** was isolated by flash column chromatography (SiO_2_, AcOEt/hexane = 1/10) in 22% yield (70 mg, 0.111 mmol) as a pale yellow solid. ^1^H NMR (400 MHz, CDCl_3_, rt, δ/ppm): 7.12 (dd, *J* = 7.8, 1.5 Hz, 2H), 6.99–6.96 (m, 4H), 6.32 (d, *J* = 7.8 Hz, 2H). ^19^F NMR (376 MHz, CDCl_3_, rt, δ/ppm): −123.5–(−123.8) (m, 2F), −140.9–(−140.9) (m, 2F), −144.5–(−144.8) (m, 2F). HRMS (DART) *m/z*: ([M + H]^+^) Calcd for C_34_H_17_F_6_N_2_S_2_^+^ 631.0732; Found 631.0733.

#### 3.2.15. 10,10’-(Perfluoro-1,4-phenylene)bis(10*H*-phenothiazine) (**4aia**)

The title compound was prepared according to General Procedure A with phenothiazine (**1a**, 100 mg, 0.50 mmol), hexafluorobenzene (**2i**, 177 μL, 1.0 mmol, 2.0 eq), and K_2_CO_3_ (277 mg, 2.0 mmol, 4.0 eq) in DMSO (5 mL) at 80 °C. **4aia** was isolated by flash column chromatography (SiO_2_, AcOEt/hexane = 1/50) in 64% yield (174 mg, 0.320 mmol) as a pale yellow solid. ^1^H NMR (400 MHz, CDCl_3_, rt, δ/ppm): 7.14 (dd, *J* = 7.3, 1.5 Hz, 2H), 7.06–6.98 (m, 4H), 6.39 (d, *J* = 8.3 Hz, 2H). ^19^F NMR (376 MHz, CDCl_3_, rt, δ/ppm): −143.0 (s, 2F). HRMS (DART) *m/z*: ([M + H]^+^) Calcd for C_30_H_17_F_4_N_2_S_2_^+^ 545.0764; Found 545.0766.

### 3.3. Sequential S_N_Ar Reaction with ***3ab***, ***3ac***, ***3af***, and ***3ag***

#### 3.3.1. 3,5-Difluoro-2,6-bis(4-methoxyphenoxy)-4-(10*H*-phenothiazin-10-yl)benzonitrile (**4abb**)

Phenothiazine derivative **3ab** (0.10 mmol), 4-methoxyphenol (0.40 mmol, 4.0 eq), and K_2_CO_3_ (0.80 mmol, 8.0 eq) were placed in a screw-capped test tube and dried under vacuum for 1 h. After backfilling with N_2_, DMF (1.5 mL) was added to the test tube. The reaction mixture was stored at room temperature for 24 h. The reaction was quenched with water (20 mL) and the mixture was transferred to a separatory funnel containing diethyl ether (20 mL). The organic layer was separated, and the aqueous layer was extracted with diethyl ether (2 × 20 mL). The combined organic fractions were washed with brine (20 mL), dried over Na_2_SO_4_, and all volatiles were removed under vacuum. **4abb** was isolated by flash column chromatography (SiO_2_, AcOEt/hexane = 1/10) in 86% yield (50 mg, 0.0862 mmol) as a pale yellow solid. ^1^H NMR (400 MHz, CDCl_3_, rt, δ/ppm): 7.06–7.03 (m, 2H), 6.99–6.83 (m, 12H), 6.20 (d, *J* = 6.8 Hz, 2H), 3.77 (s, 6H). ^19^F NMR (376 MHz, CDCl_3_, rt, δ/ppm): −132.2 (s, 2F). HRMS (DART) *m/z*: ([M + H]^+^) Calcd for C_33_H_23_F_2_N_2_S^+^ 581.1341; Found 581.1342.

#### 3.3.2. 2-(2,4,5-Trifluoro-6-nitro-3-(10H-phenothiazin-10-yl)phenyl)isoindoline-1,3-dione (**4acc**)

In a well-dried screw-capped test tube, **3ac** (78.5 mg, 0.20 mmol) was dissolved in DMF. Phthalimide (40.7 mg, 0.22 mmol, 1.1 eq) was added to the mixture and the test tube was sealed with a cap, and the reaction mixture was stirred at 60 °C for 20 h. The reaction was quenched with water (20 mL) and the mixture was then transferred to a separatory funnel with diethyl ether (20 mL). The organic layer was separated, and the aqueous layer was extracted with diethyl ether (2 × 10 mL). The combined organic fractions were washed with brine (20 mL) and then dried over Na_2_SO_4_, and all the volatiles were removed under vacuum. **4acc** was isolated by flash column chromatography (SiO_2_, AcOEt/hexane = 1/10) in 69% yield (72.3 mg, 0.139 mmol) as a white solid. ^1^H NMR (400 MHz, CDCl_3_, rt, δ/ppm): 8.00 (dd, *J* = 5.4, 2.9 Hz, 2H), 7.87 (dd, 5.9, 2.9 Hz, 2H), 7.12 (dd, *J* = 7.3, 1.5 Hz, 2H). 7.06 (dt, *J* = 7.8, 1.5 Hz, 2H), 6.98 (t, *J* = 7.6 Hz, 2H), 6.39 (d, *J* = 7.3 Hz, 2H). ^19^F NMR (376 MHz, CDCl_3_, rt, δ/ppm): −120.6–(−120.7) (m, 1F), −131.0–(−131.1) (m, 1F), −144.8–(−144.9) (m, 1F). HRMS (DART) *m/z*: ([M + H]^+^) Calcd for C_26_H_13_F_3_O_4_N_3_S^+^ 520.0573; Found 520.0572.

#### 3.3.3. 10-(2-(Benzo[*b*]thiophen-2-yl)-3,5,6-trifluoropyridin-4-yl)-10*H*-phenothiazine (**4afd**)

In a well-dried screw-capped test tube, tetrabutylammonium difluorotriphenylsilicate (TBAT, 5.4 mg, 0.01 mmol, 10 mol%) and **3af** (34.8 mg, 0.10 mmol) were added and dried under vacuum for 1 h. After backfilling with N_2_, THF (1.0 mL) and benzo[*b*]thiophen-2-yltrimethylsilane (24.8 mg, 0.12 mmol, 1.2 eq) were added to the mixture in this order. The test tube was sealed with a cap, and the reaction mixture was stirred at 60 °C for 20 h. The reaction was quenched with water (20 mL) and the mixture was then transferred to a separatory funnel with AcOEt (20 mL). The organic layer was separated, and the aqueous layer was extracted with AcOEt (2 × 10 mL). The combined organic fractions were washed with brine (20 mL) and then dried over Na_2_SO_4_, and all the volatiles were removed under vacuum. **4afd** was isolated by flash column chromatography (SiO_2_, AcOEt/hexane = 1/30) in 65% yield (30.0 mg, 0.0649 mmol) as a white solid. ^1^H NMR (400 MHz, CDCl_3_, rt, δ/ppm): 8.07 (s, 1H), 7.95–7.82 (m, 2H), 7.43–7.37 (m, 2H),7.14 (dd, *J* = 7.3, 1.5 Hz, 2H), 7.03–6.95 (m, 4H), 6.39 (d, *J* = 7.8 Hz, 2H). ^19^F NMR (376 MHz, CDCl_3_, rt, δ/ppm): −85.5–(−85.6) (m, 1F), −126.9–(−126.9) (m, 1F), −141.8–(−141.9) (m, 1F). HRMS (DART) *m/z*: ([M + H]^+^) Calcd for C_25_H_14_F_3_N_2_S_2_^+^ 463.0545; Found 463.0544.

#### 3.3.4. 10-(2,2’,3,3’,5,5’,6,6’-octafluoro-4’-(phenylethynyl)-[1,1’-biphenyl]-4-yl)-10*H*-phenothiazine (**4age**)

In a well-dried screw-capped test tube, tetrabutylammonium difluorotriphenylsilicate (TBAT, 10.8 mg, 0.02 mmol, 20 mol%) and **3ag** (51.3 mg, 0.10 mmol) were added and dried under vacuum for 1 h. After backfilling with N_2_, THF (1.0 mL) and 1-phenyl-2-(trimethylsilyl)acetylene (24 μL, 0.12 mmol, 1.2 eq) were added to the mixture in this order. The test tube was sealed with a cap, and the reaction mixture was stirred at 60 °C for 20 h. The reaction was quenched with water (20 mL) and the mixture was then transferred to a separatory funnel with AcOEt (20 mL). The organic layer was separated, and the aqueous layer was extracted with AcOEt (2 × 10 mL). The combined organic fractions were washed with brine (20 mL) and then dried over Na_2_SO_4_, and all the volatiles were removed under vacuum. **4age** was isolated by flash column chromatography (SiO_2_, AcOEt/hexane = 1/19) in 73% yield (43.3 mg, 0.0727 mmol) as a white solid. ^1^H NMR (400 MHz, CDCl_3_, rt, δ/ppm): 7.64 (dd, *J* = 8.0, 1.7 Hz, 2H), 7.47–7.42 (m, 3H), 7.12 (dd, *J* = 7.3, 1.5 Hz, 2H). 7.04 (dt, *J* = 7.8, 1.4 Hz, 2H), 6.96 (dt, *J* = 7.3, 1.5 Hz, 2H), 6.35 (d, *J* = 7.8 Hz, 2H). ^19^F NMR (376 MHz, CDCl_3_, rt, δ/ppm): −137.7–(−137.8) (m, 2F), −138.0–(−138.1) (m, 2F), −140.4–(−140.5) (m, 2F), −143.6–(−143.7) (m, 2F). HRMS (DART) *m/z*: ([M + H]^+^) Calcd for C_32_H_14_F_8_NS^+^ 596.0714; Found 596.0715.

## 4. Conclusions

In conclusion, we demonstrated a controllable S_N_Ar reaction of polyfluoroaenes with phenothiazine for the transition-metal-free synthesis of PTH derivatives. The combination of K_3_PO_4_ as the base and MeCN as the solvent was found to be widely applicable for the regioselective monosubstitution of highly reactive polyfluoroarenes, whereas the combination of K_2_CO_3_ and DMF resulted in multisubstitution. Various functional groups, including cyano, nitro, ester, and chlorine atoms, tolerated to the present conditions, thus enabling further transformations of the S_N_Ar products. The obtained fluorine-containing PTH derivatives were employed in a sequential S_N_Ar reaction to afford highly functionalized PTH derivatives. Further investigation of the optical characteristics of these compounds and their photocatalytic capabilities is currently underway.

## Figures and Tables

**Table 1:**
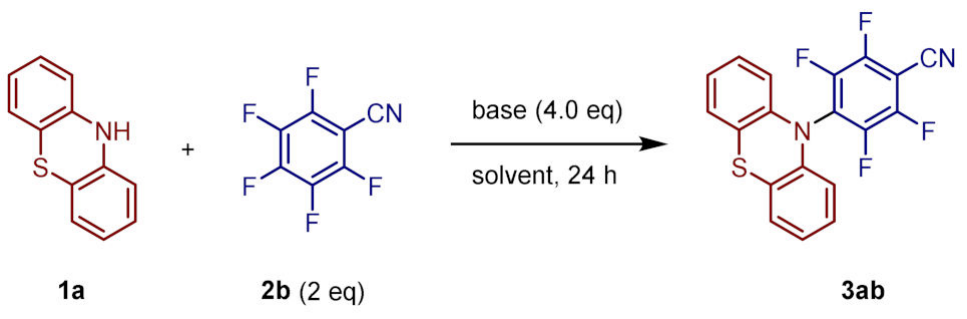
Optimization of reaction conditions using pentafluorobenzonitrile ^a^.

Entry	Base	Solvent	Temperature	3ab Yield
1	Li_2_CO_3_	DMF	60 °C	0% ^b^
2	Na_2_CO_3_	DMF	60 °C	7% ^b^
3	Cs_2_CO_3_	DMF	60 °C	13% ^b,c^
4	Li_3_PO_4_	DMF	60 °C	1% ^b^
5	Na_3_PO_4_	DMF	60 °C	13% ^b^
6	K_3_PO_4_	DMF	60 °C	48% ^d^
7	Na_3_PO_4_	DMF	80 °C	32% ^d^
8	K_3_PO_4_	DMF	80 °C	38% ^d^
9	K_3_PO_4_	MeCN	60 °C	76% ^d^
10	K_3_PO_4_	DMA	60 °C	43% ^d^
11	K_3_PO_4_	DMSO	60 °C	38% ^d^
12	K_3_PO_4_	CHCl_3_	60 °C	0%
13	K_3_PO_4_	THF	60 °C	0%
14	K_3_PO_4_	1,4-dioxane	60 °C	0%

^a^ Reaction conditions: Phenothiazine **1a** (0.50 mmol), pentafluorobenzonitrile **2b** (1.0 mmol), and base (2.0 mmol) in solvent (5.0 mL, 0.1 M). ^b^ Determined by ^19^F-NMR using 4-fluorotoluene as an internal standard. ^c^ With multi-substitution products ^d^ Isolated yield.

## Data Availability

The data presented in this study are available in Materials and Methods.

## Sample Availability

Samples of the compounds are not available from the authors.
